# Predicting Hospital Survival in Patients Admitted to ICU with Pulmonary Embolism

**DOI:** 10.1177/08850666231212875

**Published:** 2023-11-15

**Authors:** Martin J. Ryll, Aurelia Zodl, Toby N. Weingarten, Alejandro A. Rabinstein, David O. Warner, Darrell R. Schroeder, Juraj Sprung

**Affiliations:** 1 54187 Faculty of Medicine, Ludwig Maximilian University of Munich, Munich, Germany; 2 Department of Anesthesiology and Perioperative Medicine, Mayo Clinic College of Medicine and Science, Rochester, Minnesota, USA; 3 Department of Critical Care and Neurology, Mayo Clinic, Rochester, Minnesota, USA; 4 Health Sciences Research, Division of Epidemiology, Mayo Clinic College of Medicine and Science, Rochester, Minnesota, USA

**Keywords:** pulmonary embolism, intensive care unit, critical care, mortality, PESI, sPESI, ICU-sPESI

## Abstract

**Objective:**

The Pulmonary Embolism Severity Index (PESI) and simplified PESI (sPESI) predict mortality for patients with PE. We compared PESI/sPESI to the Acute Physiology and Chronic Health Evaluation IV (APACHE-IV) in predicting mortality in patients with PE admitted to the intensive care unit (ICU). Additionally, we assessed the performance of a novel ICU-sPESI score created by adding three clinical variables associated with acuity of PE presentation (intubation, confusion [altered mental status], use of vasoactive infusions) to sPESI.

**Materials and Methods:**

Using the eICU Collaborative Research Database from 2014 to 2015, we conducted a large retrospective cohort study of adult patients admitted to the ICU with a primary diagnosis of PE. We calculated APACHE-IV, PESI, sPESI, and ICU-sPESI scores and compared their performance for predicting in-hospital mortality using area under the receiver operating characteristic (AUROC) curve. Score thresholds for >99% negative predictive values (NPV) were calculated for each score. Survival was estimated using the Kaplan–Meier method.

**Results:**

We included 1424 PE cases. In-hospital mortality was 6.3% [95% CI: 5.1%-7.6%]. AUROC for APACHE-IV, PESI, and sPESI were 0.870, 0.848, and 0.777, respectively. APACHE-IV and PESI outperformed sPESI (P < 0.01 for both comparisons), while APACHE-IV and PESI demonstrated similar performance (P = 0.322). The ICU-sPESI performance was similar to APACHE-IV and PESI (AUROC = 0.847; AUROC comparison: APACHE-IV vs ICU-sPESI: P = 0.396; PESI vs ICU-sPESI: P = 0.945). Hospital mortality for ICU-sPESI scores 0-2 was 1.1%, and for scores 3, 4, 5, 6, and ≥7 was 8.6%, 11.7%, 29.2%, 37.5%, and 76.9%, respectively. Score thresholds for >99% NPV were ≤48 for APACHE-IV, ≤115 for PESI, and 0 points for sPESI and ICU-sPESI.

**Conclusions:**

By accounting for severity of PE presentation, our newly proposed ICU-sPESI score provided improved PE mortality prediction compared to the original sPESI score and offered excellent discrimination of mortality risk.

## Introduction

Pulmonary embolism (PE) is the third highest cause of cardiovascular-related death, accounting for over 100,000 annual deaths in the United States.^1^ Mortality after acute PE is between 5% and 15%,^2^ with mortality between 35% and 58% for patients presenting with hypotension and/or shock.^3^ Early establishment of mortality risk in patients with PE may play an important role in management strategies.^4,5^ The Acute Physiology and Chronic Health Evaluation IV (APACHE-IV)^6^ is one of the most advanced scoring systems designed for estimating short-term mortality among all patients admitted to an intensive care unit (ICU) using a wide range of comorbidities and clinical variables derived within the first 24 h of ICU admission.^7,8^ However, the APACHE-IV's 129 variables make its calculation complex and labor-intensive.^9,10^ In contrast, the less complex Pulmonary Embolism Severity Index (PESI), developed as a risk stratification tool for patients with PE, utilizes 11 differentially weighted variables (Supplemental Table 1).^11^ However, because PESI is considered cumbersome and is rarely calculated in clinical practice,^12^ a less complex, simplified PESI score (sPESI) was introduced, including 6 equally weighted (present =1; absent =0) and easily accessible variables (Supplemental Table 1).^13^ Interestingly, altered mental status, a variable with the highest weight in the PESI original multivariable analysis, was not included as a component of the sPESI. The PESI score demonstrated good predictive capability in identifying patients at low risk for 30-day all-cause death following PE.^6,14–16^ Although there are abundant data on the accuracy of PESI and sPESI for patients with PE, their performance has been infrequently evaluated in PE patients who were deemed to require ICU admission.^3,17^

The first aim of this study was to compare the predictive abilities of PESI and sPESI scores to the all-cause mortality APACHE-IV score using a multicenter cohort of United States patients admitted to the ICU with PE. The second aim was to test the hypothesis whether the addition of 3 early and easily accessible binary clinical markers of PE severity (Intubation, Confusion [altered mental status], Use of vasoactive infusions) to sPESI, creating a new ICU-sPESI score, could improve mortality prediction.

## Materials and Methods

### Data Source and Ethics Statement

Data were obtained from the eICU Collaborative Research Database v2.0, a multicenter critical care database provided by Philips Healthcare in collaboration with the MIT Laboratory for Computational Physiology. This database includes a wide range of clinical data for more than 200,000 ICU admissions from 208 hospitals, including both academic and community hospitals of various sizes, across all regions of the United States from 2014 and 2015.^18,19^

This study was conducted in accordance with the Declaration of Helsinki and the Strengthening the Reporting of Observational Studies in Epidemiology (STROBE) reporting guidelines.^20^ The study did not require institutional review board approval due to its retrospective nature, lack of direct patient intervention, and security framework, ensuring re-identification risks met Safe Harbor criteria as certified by Privacert (Cambridge, MA; Health Insurance Portability and Accountability Act Certification no. 1031219-2). The author Martin J. Ryll obtained access (CITI Data or Specimens Only Research Training Record ID 50703292) and was responsible for data extraction and analysis. The code for data extraction and analysis can be accessed online at github.com/RyllMartin/eICU_predicting_PE_ICU_mortality.

### Patient Selection and Data Extraction

All ICU patients with a primary admission diagnosis of PE were included, except those <18 years old or those missing key data (APACHE-IV score, survival outcome, and Glasgow Coma Scale [GCS]). Data extracted included age, sex, ethnicity, body mass index (BMI), and selected clinical variables within 24 h of admission (GCS, APACHE-IV scores, vital signs, mental status, hemodynamic management, intubation status). Other variables used to calculate the PESI and sPESI included the presence of (a) chronic lung disease (history of asthma, chronic obstructive or restrictive pulmonary disease, home oxygen requirement, or lung transplant), (b) heart failure, (c) chronic cardiopulmonary disease (history of chronic lung disease [see above], heart failure, history of myocardial infarction, coronary artery bypass, pacemaker, or implantable cardioverter-defibrillator), and (d) cancer history. Variables for heart rate, body temperature, and respiratory rate were derived from the Acute Physiology Score (APS; part of APACHE) vitals. Missing values among the vitals variables were assumed as normal, in line with the original PESI publication.^11^ Altered mental status (AMS) or confusion was deemed present if the GCS category verbal response was <5 (i.e., not verbally responsive and oriented). The final PESI and sPESI risk scores were calculated from these components as listed in Supplemental Table 1.^11,13^ For the ICU-sPESI, a 9-variable equally weighted score was designed by adding 3 binary clinical variables of disease acuity to the sPESI if present within the first 24 h after admission: (a) respiratory failure requiring intubation (I), (b) confusion (C) or altered mental status; and (c) need for use (U) of vasopressor or inotropic support. These markers were chosen based on prior studies showing their independent association with PE mortality.^3,21,22^

### Statistical Analyses

Vital signs were assessed from medians calculated over 30 min and we used the most extreme values for the final score calculation. Continuous data are presented as median and IQR, categorical and binary variables as count and percentage. Categorical and binary variables were compared between survivors and non-survivors by Chi-Square test or Fisher-exact test as appropriate. Continuous variables were tested for normal distribution via Shapiro–Wilk test and compared by independent t-test or Mann–Whitney U test where appropriate. We derived mortality predictions for each score using a univariate logistic regression model with the score as the independent and in-hospital mortality as the dependent variable. Receiver operating characteristic (ROC) curves were calculated and plotted for the four scores. The areas under the receiver operating characteristic (AUROCs) curve of the scores were compared with a test for comparing AUROCs on the same patient cohort as described by Hanley and McNeil.^23^ For the main analysis, PESI, sPESI, and ICU-sPESI were calculated using data from the first 24 h following ICU admission. We performed a sensitivity analysis that examined AUROC for PESI, sPESI, and ICU-sPESI calculated at different time points following ICU admission (2, 3, 6, and 12 h after admission to the ICU). In addition, we calculated the highest score thresholds associated with negative predictive values (NPV) exceeding 99%. To visualize the fit of our models at different score ranges, the predicted and actual in-hospital mortality were plotted against deciles of the APACHE-IV and PESI scores and against direct score value for the sPESI and ICU-sPESI scores. Using the Kaplan–Meier method, survival curves were calculated for different patient groups stratified by PESI classes, sPESI scores, and ICU-sPESI scores. All data were censored at 40 days post-ICU admission, with the last event recorded on the 39^th^ day. Patients who survived to hospital discharge were assumed alive post-discharge. To assess potential differences among these survival curves, a multivariate log-rank test was first utilized. In the case of observed differences, pairwise log-rank tests were performed, adjusting for multiple comparisons using the Bonferroni correction. In addition, Kaplan-Meier survival curves with 95% confidence intervals were created for 4 ICU-sPESI risk classes: Class I (ICU-sPESI score ≤2 points), Class II (3-4 points), Class III (5-6 points), and Class IV (≥7 points). The class groupings were based on pairwise comparisons of individual score survival curves (unadjusted log-rank test) with groups created for scores whose survival curves did not differ significantly. All reported tests were two-sided, and P-values < 0.05% were considered statistically significant. Statistical analyses were performed with the Python v.3.9 (Python Software Foundation, Wilmington, Delaware, USA) program.

## Results

Of the 1697 patients with a primary admission diagnosis of PE, 270 were excluded due to missing key data and 3 patients were under the age of 18 years. The study cohort comprised the remaining 1424 patients of which 90 died in the hospital yielding a mortality rate of 6.3% [95% CI: 5.1%-7.6%]. Median [IQR] time to all-cause death for 1424 patients was 3.7 [1.2-9.1] days. For survivors, hospital length of stay was 5.7 [3.6-8.6] days, and ICU length of stay was 1.8 [1.0-2.8] days.

Compared to survivors, nonsurvivors were older (median age 70 vs 62 years, P < 0.001) and had higher rates of cancer history (34.4% vs 16.1%, P < 0.001), obstructive pulmonary disease (26.7% vs 16.1%, P = 0.014), dementia (7.8% vs 2.5%, P = 0.011) and liver cirrhosis (4.4% vs 0.8%, P = 0.012) (Table 1). Fewer nonsurvivors reported a history of previous thromboembolic events than survivors, but the difference did not reach significance (7.8% vs 15.3%, P = 0.074). The rates of thrombolytic use were low and not significantly different between survivors and nonsurvivors (3.8% vs 2.2%, respectively P = 0.770). A detailed presentation of comorbidities among the two groups are presented in Supplemental Table 2.

**Table 1.** Demographics and Comorbidities among Patients with a Primary Diagnosis of Pulmonary Embolism Requiring Intensive Care Unit Admission.

Characteristics	All patients ( N = 1424)	Survivors ( N = 1334)	Non-survivors ( N = 90)	P value
**Demographics**				
Age, years	63 [51-75]	62 [51-74]	70 [59-81]	< 0.001
Age <60	617 (43.3%)	592 (44.4%)	25 (27.8%)	
Age 61-70	338 (23.7%)	316 (23.7%)	22 (24.4%)	
Age 71-80	262 (18.4%)	242 (18.1%)	20 (22.2%)	
Age >80	207 (14.5%)	184 (13.8%)	23 (25.6%)	
Sex (male)	710 (49.9%)	670 (50.2%)	40 (44.4%)	0.341
Body mass index, kg/m^2^	31.3 [25.9-37.6]	31.4 [26.1-37.7]	27.2 [23.0 −33.7]	< 0.001
**Ethnicity**				0.098
Caucasian	1120 (78.7%)	1042 (78.1%)	78 (86.7%)	
African American	179 (12.6%)	174 (13.0%)	5 (5.6%)	
Other	125 (8.8%)	118 (8.8%)	7 (7.8%)	
**Comorbidities**				
**Cardiovascular**				
Hypertension	670 (47.1%)	628 (47.1%)	42 (46.7%)	1.00
Prior venous thrombosis/PE	211 (14.8%)	204 (15.3%)	7 (7.8%)	0.074
Coronary artery disease ^a^	211 (14.8%)	196 (14.7%)	15 (16.7%)	0.721
Congestive heart failure	116 (8.1%)	108 (8.1%)	8 (8.9%)	0.947
Cardiac dysrhythmias	121 (8.5%)	111 (8.3%)	10 (11.1%)	0.469
Pacemaker/AICD	26 (1.8%)	22 (1.6%)	4 (4.4%)	0.077
Cardiac valvular disease	22 (1.5%)	21 (1.6%)	1 (1.1%)	1.00
**Pulmonary**				
Obstructive lung disease	239 (16.8%)	215 (16.1%)	24 (26.7%)	0.014
Restrictive lung disease	13 (0.9%)	12 (0.9%)	1 (1.1%)	0.574
Home oxygen therapy	35 (2.5%)	30 (2.2%)	5 (5.6%)	0.108
**Neurologic conditions**				
Seizures	51 (3.6%)	50 (3.7%)	1 (1.1%)	0.37
Dementia	41 (2.9%)	34 (2.5%)	7 (7.8%)	0.011
Neuromuscular disease	9 (0.6%)	7 (0.5%)	2 (2.2%)	0.106
Intracranial mass	5 (0.4%)	5 (0.4%)	0 (0%)	1.00
**Oncologic/hematologic**				
Cancer	246 (17.3%)	215 (16.1%)	31 (34.4%)	< 0.001
Anemias	11 (0.8%)	10 (0.7%)	1 (1.1%)	0.514
Sickle cell disease	7 (0.5%)	7 (0.5%)	0 (0%)	1.00
**Other**				
Diabetes mellitus	284 (19.9%)	263 (19.7%)	21 (23.3%)	0.487
Thyroid disorders	143 (10.0%)	133 (10.0%)	10 (11.1%)	0.867
Liver cirrhosis	15 (1.1%)	11 (0.8%)	4 (4.4%)	0.012
Renal insufficiency	55 (3.9%)	48 (3.6%)	7 (7.8%)	0.087

Binary variables and categories are shown as count (percentage), continuous variables as median [25th quantile-75th quantile]. Individual features had no missing values, except for body mass index with 3.7% missing values and ethnicity with <1% missing values.

^a^ Includes other major vessel atherosclerotic disease.

Abbreviations: ITP, idiopathic thrombocytopenic purpura; AICD, automatic implantable cardioverter defibrillator; PE, pulmonary embolism.

Supplemental Table 3 shows PESI, sPESI, and ICU-sPESI score components on admission according to outcome (survival vs no-survival). Except for sex and history of heart failure, all individual components of PESI and sPESI were significantly associated with in-hospital mortality. Regarding the three additional ICU-sPESI variables, nonsurvivors were more likely to be intubated, have altered mental status, and to be hemodynamically unstable requiring vasoactive support (all P < 0.001). Additional vital signs for survivors and nonsurvivors obtained within 24 h of ICU admission are provided in Supplemental Table 4.

Components of the PESI, sPESI, and ICU-sPESI scores and their distribution on admission are shown in Table 2. Compared to survivors, non-survivors had significantly higher APACHE-IV (80 vs 40, P < 0.001), PESI (191 vs 118, P < 0.001), sPESI (3 vs 2, P < 0.001), and ICU-sPESI (4 vs 2, P < 0.001) scores. For all scores, mortality increased as the score increased.

**Table 2.** APACHE-IV, PESI, sPESI, and ICU-sPESI Scores Obtained Within 24 h Following Admission to the ICU.

Scores	All patients ( N = 1424)	Survivors ( N = 1334)	Nonsurvivors ( N = 90)	Actual mortality	P values
**APACHE-IV score**	42 [31-55]	40 [30-52]	80 [56-101]	—	<0.001
**PESI score**	121 [93-154]	118 [92-148]	191 [157-217]	—	<0.001
*PESI risk classes* ^a^					<0.001
Class I	98 (6.9%)	98 (7.3%)	0 (0%)	[0.0%]	
Class II	160 (11.2%)	158 (11.8%)	2 (2.2%)	[1.2%]	
Class III	256 (18.0%)	253 (19.0%)	3 (3.3%)	[1.2%]	
Class IV	248 (17.4%)	244 (18.3%)	4 (4.4%)	[1.6%]	
Class V	662 (46.5%)	581 (43.6%)	81 (90.0%)	[12.2%]	
**sPESI score**	2.0 [1.0-3.0]	2.0 [1.0-3.0]	3.0 [2.0-4.0]	—	<0.001
*sPESI points* ^b^				—	<0.001
0	177 (12.4%)	176 (13.2%)	1 (1.1%)	[0.6%]	
1	403 (28.3%)	395 (29.6%)	8 (8.9%)	[2.0%]	
2	438 (30.8%)	420 (31.5%)	11 (20.0%)	[4.1%]	
3	255 (19.9%)	229 (17.2%)	26 (28.9%)	[10.2%]	
4	121 (8.5%)	93 (7.0%)	28 (31.1%)	[23.1%]	
5	30 (2.1%)	21 (1.6%)	9 (10.0%)	[30.0%]	
**ICU-sPESI score**	2.0 [1.0-3.0]	2.0 [1.0-3.0]	4.0 [3.0-5.0]	—	<0.001
*ICU-sPESI points*				—	<0.001
0	168 (11.8%)	167 (12.5%)	1 (1.1%)	[0.6%]	
1	365 (25.6%)	360 (27.0%)	5 (5.6%)	[1.4%]	
2	393 (27.6%)	389 (29.2%)	4 (4.4%)	[1.0%]	
3	244 (17.1%)	223 (16.7%)	21 (23.3%)	[8.6%]	
4	137 (9.3%)	121 (9.1%)	16 (17.8%)	[11.7%]	
5	72 (5.1%)	51 (3.8%)	21 (23.3%)	[29.2%]	
6	32 (2.2%)	20 (1.5%)	12 (13.3%)	[37.5%]	
≥7 ^c^	13 (0.9%)	3 (0.2%)	10 (11.1%)	[76.9%]	

Binary variables are shown as count and percentage of patient ICU admissions, continuous variables (scores) as median [IQR]. There were no missing values.

^a^ PESI score classes: Class I, ≤65 (very low risk); Class II, 66-85 (low risk); Class III, 86-105 (intermediate risk); Class IV, 106-125 (high risk); Class V, >125 (very high risk). Generally, Class I and II are considered “low-risk”; Classes III-V, “high risk”.^11,13^

^b^ sPESI score is generally considered “low risk” if 0; and high risk if ≥1.^11,13^

^c^ Only 1 patient had an ICU-sPESI score of 8, and 0 patients had an ICU-sPESI score of 9.

Abbreviations: Acute Physiology and Chronic Health Evaluation; PESI, Pulmonary Embolism Severity Index; sPESI, simplified Pulmonary Embolism Severity Index; ICU-sPESI, ICU-modified simplified Pulmonary Embolism Severity Index; ICU, intensive care unit.

**Figure 1.**
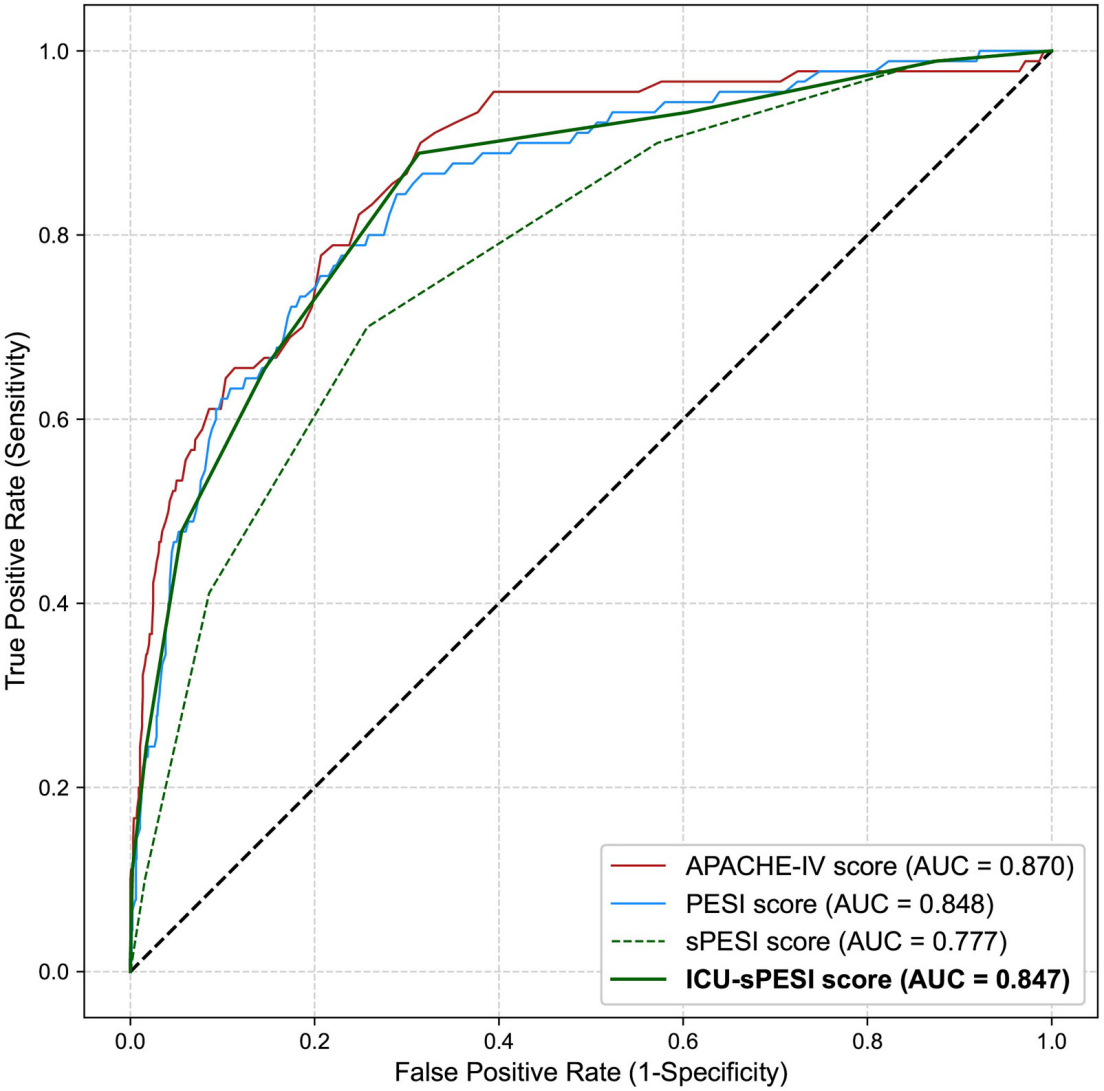
Receiver operating characteristic curves for the APACHE-IV, PESI, sPESI, and ICU-sPESI score for predicting in-hospital mortality.

ROC curves were used to assess the performance of the four scores in regard to prediction of mortality. The AUROC for APACHE-IV calculated from data available at 24 h after admission was 0.870, for PESI 0.848, and for sPESI 0.770 (Figure 1). Numerically, APACHE-IV had the highest discriminative ability among the three scoring systems; however, its AUROC was not significantly higher than that of the PESI (P = 0.322). Both the APACHE-IV and PESI outperformed sPESI, as evidenced by the differences in their AUROCs (sPESI vs APACHE-IV, P = 0.006, sPESI vs PESI, P = 0.010). The ICU-sPESI had an AUROC of 0.847, which was greater than the sPESI (P < 0.001) and comparable to APACHE-IV and PESI (APACHE-IV vs ICU-sPESI: P = 0.396; PESI vs ICU-sPESI: P = 0.945, Figure 1). The sensitivity analysis comparing AUROCs for PE risk scores at shorter time intervals (2, 3, 6, 12 h) after ICU admission demonstrated slightly lower AUROC values, but the pattern was similar for all three risk scores (Supplemental Table 5). Using “any type of ventilation” (invasive and/or noninvasive) instead of “intubation/invasive mechanical ventilation” for the ICU-sPESI's “I” variable did not alter the ICU-sPESI's predictive performance (Supplemental Figure 2).

**Figure 2.**
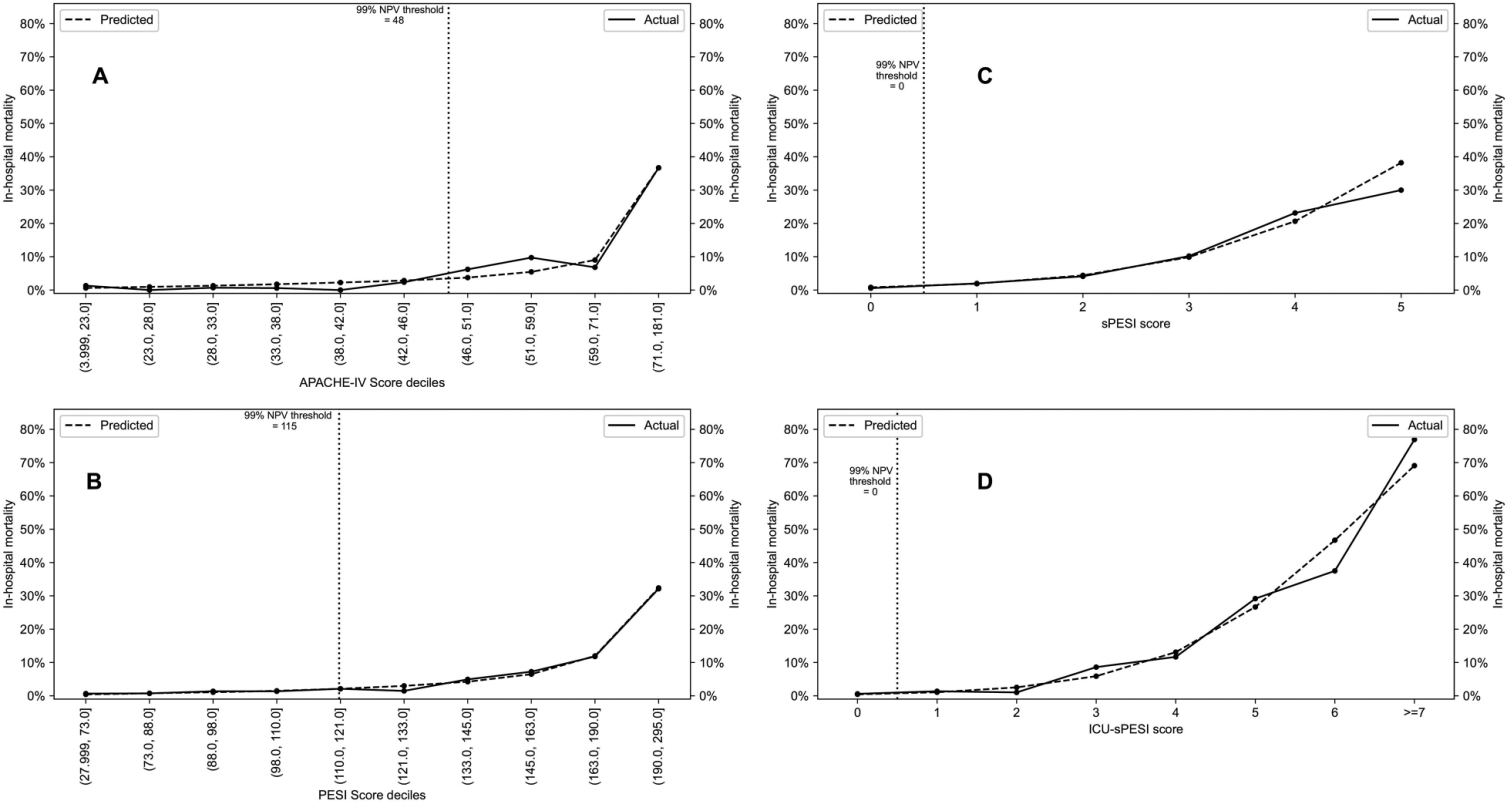
(A-D) Actual versus predicted mortality across the spectrum of scores for APACHE-IV (A), PESI (B) (both scores shown in deciles), sPESI (C), and ICU-sPESI (D) (both scores shown in direct score values). Vertical dotted line indicates the score threshold for a NPV of >99% for each test for critically ill patients with PE.

Figure 2 depicts the observed and predicted in-hospital mortality from the univariate logistic regression models for APACHE-IV, PESI, sPESI, and ICU-sPESI. All scores showed a comparable observed and predicted in-hospital mortality across their respective score ranges, demonstrating accurate calibration for the logistic regression models. To evaluate the predictive capability for low mortality risk, the highest (best) score thresholds for >99% NPV were calculated. The corresponding score thresholds were ≤48 for APACHE-IV, ≤115 for PESI, and zero (0) points for sPESI and ICU-sPESI (Figure 2 vertical dotted lines).

**Figure 3.**
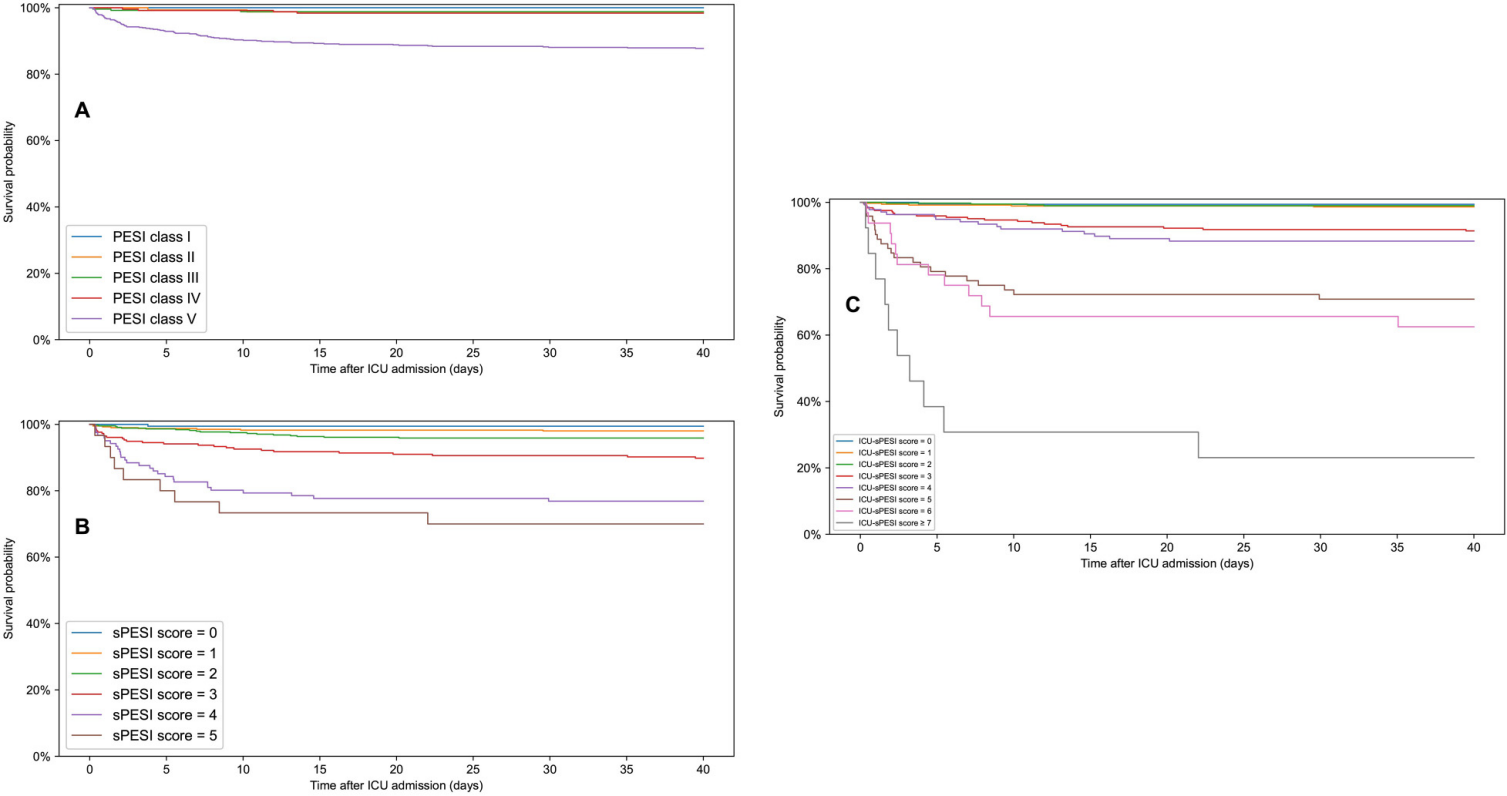
Kaplan–Meier survival curves following ICU admission for PESI classes (A), sPESI scores (B), and ICU-sPESI scores (C). For all scores, survival differed significantly across all groups (multivariate log-rank test; P < 0.001). Pairwise survival curve comparisons statistically significant by log-rank test (adjusted for multiple comparisons by Bonferroni correction): (A) PESI classes: PESI class V versus IV, III, II, I: P-values <0.001, <0.001, <0.001, 0.004, respectively. (B) sPESI score: sPESI score 5 versus 3, 2, 1, 0: P-values 0.015, <0.001, <0.001, <0.001, respectively. sPESI score 4 versus 3, 2, 1, 0: P-values 0.010, <0.001, <0.001, <0.001, respectively. sPESI score 3 versus 2, 1, 0: P values 0.021, <0.001, 0.010, respectively. (C) ICU-sPESI score: ICU-sPESI score ≥7 versus 5, 4, 3, 2, 1, 0: P-values 0.005, <0.001, <0.001, <0.001, <0.001, <0.001, respectively. ICU-sPESI score 6 versus 4, 3, 2, 1, 0: P-values 0.004, <0.001, <0.001, <0.001, <0.001, respectively. ICU-sPESI score 5 versus 4, 3, 2, 1, 0: P-values 0.027, <0.001, <0.001, <0.001, <0.001, respectively. ICU-sPESI score 4 versus 2, 1, 0: All P values <0.001. ICU-sPESI score 3 versus 2, 1, 0: P-values <0.001, <0.001, 0.011 respectively.

**Figure 4.**
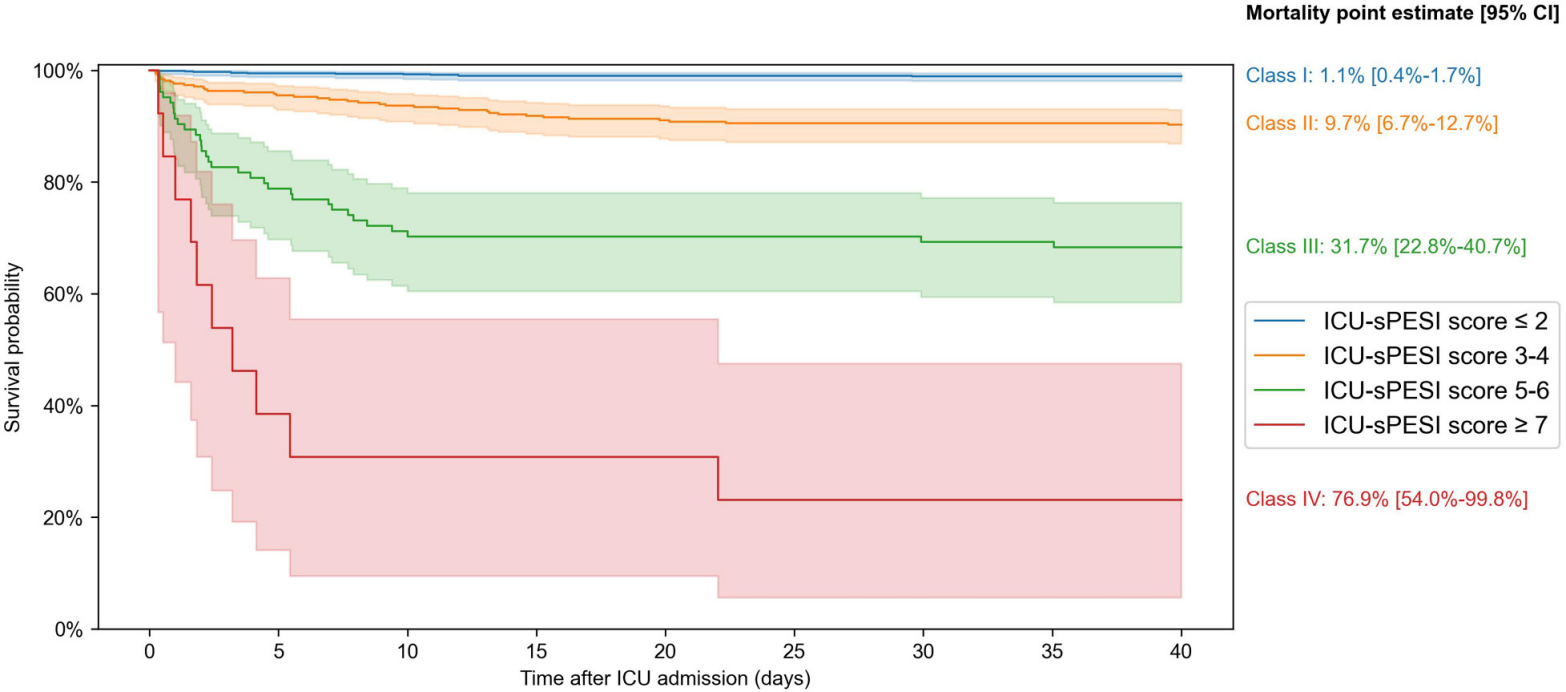
Kaplan–Meier survival curves with 95% confidence intervals for the proposed four ICU-sPESI risk classes: Class I (ICU-sPESI score ≤2), Class II (3-4), Class III (5-6), and Class IV (≥7). The class groupings were based on pairwise comparisons of individual score survival curves (log-rank test) with groups created for scores whose survival curves did not differ significantly. No significant differences were found between scores 0 versus 1 (P = 0.431), 0 versus 2 (P = 0.627), 1 versus 2 (P = 0.653), 3 versus 4 (P = 0.336), and 5 versus 6 (P = 0.472). The grouping of scores ≥7 was done due to the limited patient numbers with ICU-sPESI scores of 8 (n = 1) and 9 (n = 0). A multivariate log-rank test confirmed a significant overall difference across the four risk classes (P < 0.001). Point estimates [95% CI] for all-cause in-hospital mortality in the four ICU-sPESI risk classes are provided in the figure.

Figure 3 shows the Kaplan–Meier survival plots for PESI classes, and sPESI and ICU-sPESI scores. For all scores, survival differed across groups and decreased as the scores increased (log-rank test P < 0.001). Details regarding statistically significant pairwise comparisons between respective score groups are provided in the footnote of Figure 3. Figure 4 shows Kaplan–Meier curves with 95% confidence intervals for the estimated survival rate when ICU-sPESI scores were grouped into 4 risk classes (see Methods). Hospital mortality increased in each higher ICU-sPESI risk class (point estimate [95% CI]) for Class I 1.1% [0.4%-1.7%]; Class II 9.7% [6.7%-12.7%]; Class III 31.7% [22.8%-40.7%]; and Class IV, 76.9% [54.0%-99.8%].

## Discussion

For patients with PE admitted to the ICU, the APACHE-IV and PESI provided comparable predictive power for in-hospital mortality, whereas the discriminative predictive power of sPESI was lower. Thus, by incorporating three easily obtainable markers of PE acuity into the sPESI score, we created a novel ICU-sPESI score that allowed for improved discrimination of PE mortality risk.

For patients admitted to ICU with PE the APACHE-IV AUROC was 0.870, similar to that observed in the original validation study of critically ill adults (0.880).^6^ The PESI and sPESI are validated assessments for mortality prediction from PE in patients.^13,24,25^ Although in our study the AUROC of sPESI (0.770) for prediction of in-hospital mortality was inferior to that of PESI (0.848), both tests performed better than in reports by Barnes^26^ for 7- and 30-day mortality (PESI 0.652, sPESI 0.666), and İdin^17^ (PESI 0.624) for 30-day mortality. The discrepancy in AUROCs between our study and that of Barnes^26^ and İdin^17^ is not clear. Multiple reports demonstrated that sPESI is sensitive in identifying low-risk PE patients.^12,13,15,24,25^ Yet, in our study of PE patients admitted to the ICU, sPESI had inferior predictive performance compared to PESI, possibly reflecting the exclusion of variable such as altered mental status which had the highest weight in PESI score. To improve the accuracy of both PESI and sPESI, the European Society of Cardiology (ESC)^27^ recommended factoring in parameters such as serum biomarkers (brain natriuretic peptide or troponin), radiologic and echocardiographic indicators of right ventricular dysfunction.^28–30^ While this approach may improve the score performance, it introduces complexity to calculation. The eICU database lacks imaging data and did not report markers of myocardial injury or dysfunction for >60% of our patients, thereby limiting our ability to evaluate the impact of the ESC-recommended^27^ variables.

It is important to be able to identify low-risk PE patients early, as this may help with triaging and stewardship of resources.^11,15,16,28,31–33^ However, defining a score that precisely identifies low-risk PE may be challenging. Based on 99% NPV calculations, the low-risk PE patients in our study were those with an APACHE-IV score ≤48, a PESI score <115, and sPESI and ICU-sPESI scores of zero points. However, some studies reported different cutoff points for identifying low-risk PE.^15^,^34^ For example, Brikho^15^ and Bellou^34^ proposed a PESI score of <85 (PESI class ≤ II) as a cutoff point for outpatient treatment, despite associated 30-day mortality of 1.6%-3.5%. In contrast, a higher PESI score of ≤115 (Class IV, i.e., high-risk PESI score)^15^ in our study was associated with very low (0.93%) mortality rate, and therefore may be considered a low PE risk. These discrepancies in cutoff points of low-risk PE may be related to study designs, definitions of timing of death (in-hospital, vs 7-day, vs 30-day death), type of cohort examined (eg, all patients vs ICU admissions) or due to reports coming from single institution with nonstandard expertise in managing PE.

It is also important to develop a reliable and simple score that predicts risk of high PE mortality. Since high mortality risk was not precisely discriminated by PESI classes and sPESI score, we postulated that adding easily obtainable variables related to the severity of clinical presentation may improve the ability of the sPESI to better categorize mortality risk in PE patients admitted to the ICU. Specifically, in earlier reports the use of vasopressors and respiratory insufficiency requiring intubation/mechanical ventilation were predictors of mortality in PE patients.^3,21^ Furthermore, altered mental status was an independent predictor [OR 9.1] of a life-threatening adverse outcomes in PE patients.^22^ Using this information, we proposed a new ICU-sPESI mortality prediction score by adding these clinical variables to the original sPESI score. This novel score demonstrated improved discrimination of mortality risk compared to PESI and sPESI.

### Strengths and Limitations

Because our study is based on data from a large multicenter registry, it measures mortality across institutions with diverse patients and different expertise in managing PE, contributing to external validity. However, using this database leads to certain limitations. In our study, we included patients with a primary diagnosis of PE on admission to the ICU rather than diagnosis confirmed on discharge because the eICU does not report a final discharge diagnosis or imaging data. Therefore, we cannot exclude potential misdiagnosis of PE on admission to the ICU. Furthermore, we were unable to compare our findings to patients with PE admitted to other services, such as regular wards, as data on these patients are not available in the eICU. Finally, in our study, the overall in-hospital PE mortality (6.3%) was slightly lower than in a previous US-based large multicenter study on ICU admissions of patients with PE (9.2%-9.5%).^35^ Sauer et al.^36^ examined data from four adult clinical ICU datasets which also included the eICU Collaborative Research Database. They found that the four databases differ in sample sizes, acuity of illness for admitted patients, treatment intensities, and frequency of reported parameters. Therefore, it is possible that outcomes reported from different databases may differ due to differences in patient acuity and treatment intensities.

We used vital signs and clinical events observed during 24 h after ICU admission as score components of PE severity. Practical predictive scores would be most useful if obtained at shorter timeframes after admission, therefore further studies should examine this prospectively. However, our sensitivity analysis of AUROCs for PESI, sPESI, and ICU-sPESI at various time periods during the first 24 h following ICU admission showed comparable albeit slightly diminished predictive accuracy, mainly attributable to the clinical events. As data on these clinical events is considerably less granular, this may be explained by the lag between event occurrence and actual event charting, or alternatively by the 24-h period allowing more time to establish symptomatology of PE relevant for prediction of outcome. Although our cohort consisted of PE patients admitted to the ICU, we do not have insight of true indications for ICU admission, and literature has shown that ICU admissions for PE have a huge variation between hospitals with differing acuity thresholds for ICU admission criteria.^35^ Furthermore, as our results represent aggregates of the data of many centers, the predictive performance of the scores may not be generalizable to a center where the level of expertise and treatment modalities substantially fall outside the average standards of care for PE. Specifically, it has been shown that patients with acute PE may benefit when treated at high-volume centers by experienced physicians.^37,38^ In addition, the reported accuracy in predicting mortality using risk scores is associated with advances in treatments.^39^ Specifically, mortality in high-risk patients with PE decreased from 1999 to 2017, primarily due to improved treatment of most severe PE patients with shock and cardiac arrest^39^ which underscores the need to continue reassessing the performance of these scores over time.

## Conclusion

In conclusion, while APACHE-IV and PESI scores demonstrated good prediction of mortality among critically ill patients with PE, the performance of sPESI was lower. After incorporating three clinical markers of disease acuity into the sPESI calculation, the discriminative ability of the novel ICU-sPESI score improved and was not different from that of PESI. ICU-sPESI scores provide improved prediction of mortality among high-risk PE patients admitted to the ICU. Future prospective studies are needed to validate the accuracy of the ICU-sPESI score for PE patients admitted to the ICU, especially for those considered to be at high risk.

## Supplemental Material

sj-docx-1-jic-10.1177_08850666231212875 - Supplemental material for Predicting Hospital Survival in Patients Admitted to ICU with Pulmonary Embolism Supplemental material, sj-docx-1-jic-10.1177_08850666231212875 for Predicting Hospital Survival in Patients Admitted to ICU with Pulmonary Embolism by Martin J. Ryll, Aurelia Zodl, Toby N. Weingarten, Alejandro A. Rabinstein, David O. Warner, Darrell R. Schroeder and Juraj Sprung in Journal of Intensive Care Medicine

sj-docx-2-jic-10.1177_08850666231212875 - Supplemental material for Predicting Hospital Survival in Patients Admitted to ICU with Pulmonary Embolism Supplemental material, sj-docx-2-jic-10.1177_08850666231212875 for Predicting Hospital Survival in Patients Admitted to ICU with Pulmonary Embolism by Martin J. Ryll, Aurelia Zodl, Toby N. Weingarten, Alejandro A. Rabinstein, David O. Warner, Darrell R. Schroeder and Juraj Sprung in Journal of Intensive Care Medicine

sj-docx-3-jic-10.1177_08850666231212875 - Supplemental material for Predicting Hospital Survival in Patients Admitted to ICU with Pulmonary Embolism Supplemental material, sj-docx-3-jic-10.1177_08850666231212875 for Predicting Hospital Survival in Patients Admitted to ICU with Pulmonary Embolism by Martin J. Ryll, Aurelia Zodl, Toby N. Weingarten, Alejandro A. Rabinstein, David O. Warner, Darrell R. Schroeder and Juraj Sprung in Journal of Intensive Care Medicine

sj-docx-4-jic-10.1177_08850666231212875 - Supplemental material for Predicting Hospital Survival in Patients Admitted to ICU with Pulmonary Embolism Supplemental material, sj-docx-4-jic-10.1177_08850666231212875 for Predicting Hospital Survival in Patients Admitted to ICU with Pulmonary Embolism by Martin J. Ryll, Aurelia Zodl, Toby N. Weingarten, Alejandro A. Rabinstein, David O. Warner, Darrell R. Schroeder and Juraj Sprung in Journal of Intensive Care Medicine

sj-docx-5-jic-10.1177_08850666231212875 - Supplemental material for Predicting Hospital Survival in Patients Admitted to ICU with Pulmonary Embolism Supplemental material, sj-docx-5-jic-10.1177_08850666231212875 for Predicting Hospital Survival in Patients Admitted to ICU with Pulmonary Embolism by Martin J. Ryll, Aurelia Zodl, Toby N. Weingarten, Alejandro A. Rabinstein, David O. Warner, Darrell R. Schroeder and Juraj Sprung in Journal of Intensive Care Medicine

sj-docx-6-jic-10.1177_08850666231212875 - Supplemental material for Predicting Hospital Survival in Patients Admitted to ICU with Pulmonary Embolism Supplemental material, sj-docx-6-jic-10.1177_08850666231212875 for Predicting Hospital Survival in Patients Admitted to ICU with Pulmonary Embolism by Martin J. Ryll, Aurelia Zodl, Toby N. Weingarten, Alejandro A. Rabinstein, David O. Warner, Darrell R. Schroeder and Juraj Sprung in Journal of Intensive Care Medicine

sj-docx-7-jic-10.1177_08850666231212875 - Supplemental material for Predicting Hospital Survival in Patients Admitted to ICU with Pulmonary Embolism Supplemental material, sj-docx-7-jic-10.1177_08850666231212875 for Predicting Hospital Survival in Patients Admitted to ICU with Pulmonary Embolism by Martin J. Ryll, Aurelia Zodl, Toby N. Weingarten, Alejandro A. Rabinstein, David O. Warner, Darrell R. Schroeder and Juraj Sprung in Journal of Intensive Care Medicine

## Appendices

### Appendix A—Abbreviations

APACHE-IV: Acute Physiology and Chronic Health Evaluation IV
APS: Acute Physiology Score
AUROC: Area Under the Receiver Operating Curve
BMI: Body Mass Index
CITI: Collaborative Institutional Training Initiative
eICU: Electronic Intensive Care Unit
GCS: Glasgow Coma Scale
ICU: Intensive Care Unit
IQR: Interquartile Range
ICU-sPESI: Intensive Care Unit-Simplified Pulmonary Embolism Severity Index
MIT: Massachusetts Institute of Technology
PE: Pulmonary Embolism
PESI: Pulmonary Embolism Severity Index
ROC: Receiver Operating Characteristic
sPESI: Simplified Pulmonary Embolism Severity Index
STROBE: Strengthening the Reporting of Observational Studies in Epidemiology
