# Supplementary figures and images for: Predicting Hospital Survival in Patients Admitted to ICU with Pulmonary Embolism
Source: J Intensive Care Med. 2023 Nov 15;39(5):455–64. doi: 10.1177/08850666231212875 (PMC10935623; doi:10.1177/08850666231212875)

**Supplementary Figure 1.** Cumulative mortality after admission to the ICU.

**
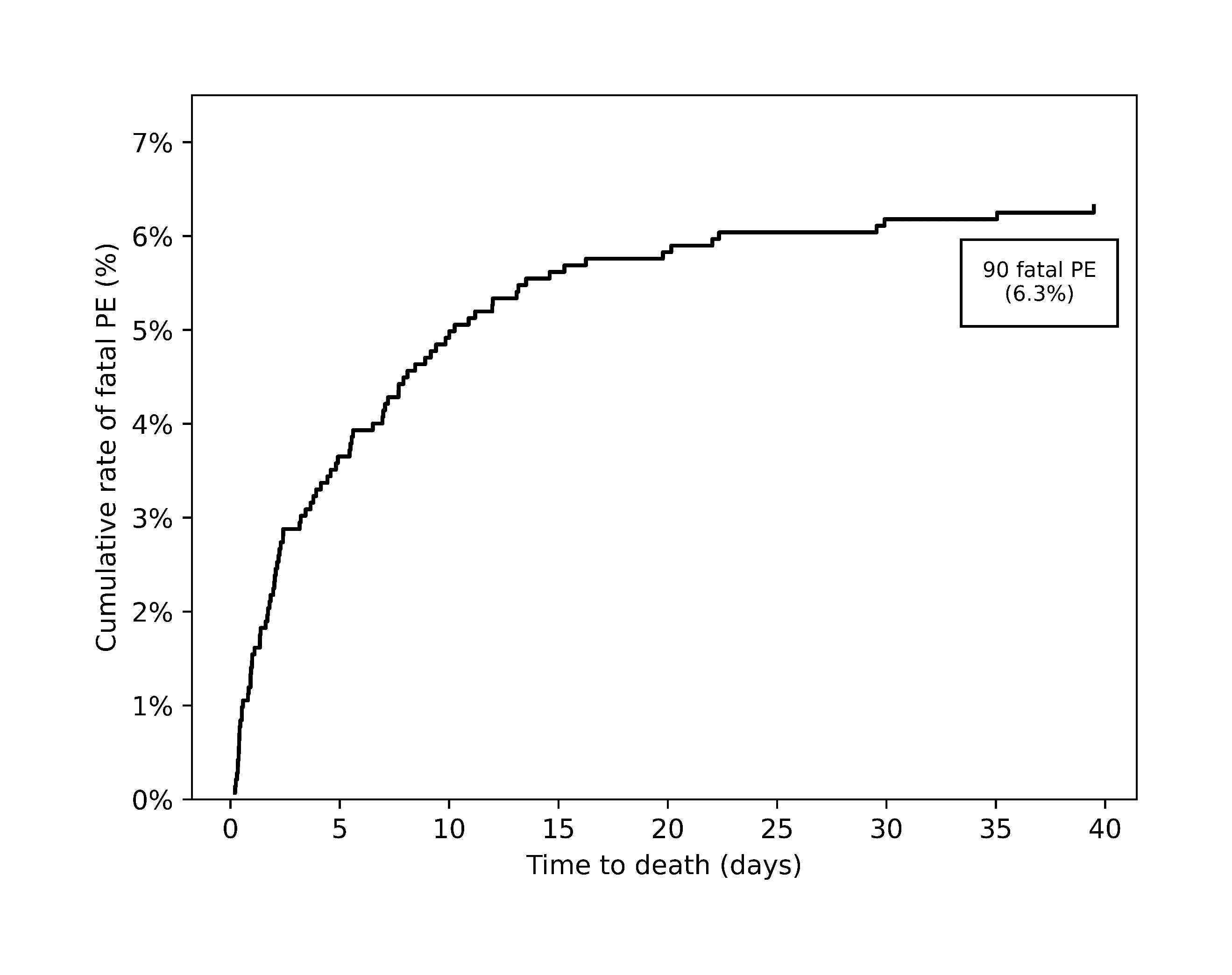
**

Supplement: sj-docx-1-jic-10.1177_08850666231212875 - Supplemental material for Predicting Hospital Survival in Patients Admitted to ICU with Pulmonary Embolism [file sj-docx-1-jic-10.1177_08850666231212875.docx]
