# Supplementary material for: Predicting Hospital Survival in Patients Admitted to ICU with Pulmonary Embolism
Source: J Intensive Care Med. 2023 Nov 15;39(5):455–64. doi: 10.1177/08850666231212875 (PMC10935623; doi:10.1177/08850666231212875)
Supplement: sj-docx-2-jic-10.1177_08850666231212875 - Supplemental material for Predicting Hospital Survival in Patients Admitted to ICU with Pulmonary Embolism [file sj-docx-2-jic-10.1177_08850666231212875.docx]

**
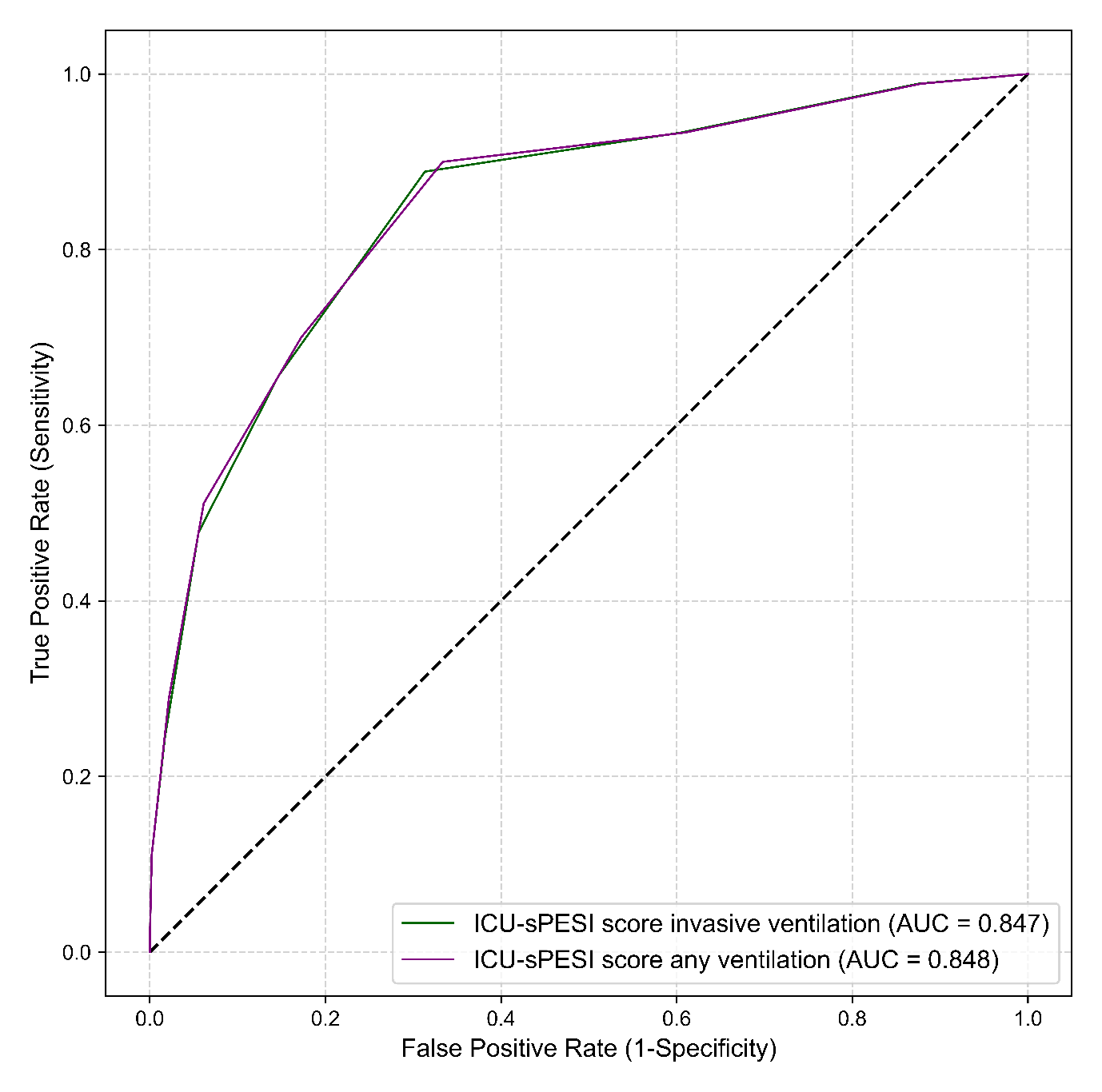
Supplementary Figure 2.** ROC curves for ‘Any type of ventilation’ vs ‘Intubation/invasive mechanical ventilation’ as ICU-sPESI’s ‘I’ variable
