# Supplementary material for: Predicting Hospital Survival in Patients Admitted to ICU with Pulmonary Embolism
Source: J Intensive Care Med. 2023 Nov 15;39(5):455–64. doi: 10.1177/08850666231212875 (PMC10935623; doi:10.1177/08850666231212875)
Supplement: sj-docx-3-jic-10.1177_08850666231212875 - Supplemental material for Predicting Hospital Survival in Patients Admitted to ICU with Pulmonary Embolism [file sj-docx-3-jic-10.1177_08850666231212875.docx]

**Supplementary Table 1.** Variables included in the Pulmonary Embolism Severity Index (PESI)^10^ and Simplified Pulmonary Embolism Severity Index (sPESI)^12^ and three variables of clinical acuity added to the sPESI to create the ICU-modified simplified Pulmonary Embolism Severity Index (ICU-sPESI).

| **PESI** | **Points** |
| --- | --- |
| Age, in years | eg, 75 |
| Male sex | 10 |
| History of cancer | 30 |
| History of heart failure | 10 |
| History of chronic lung disease | 10 |
| Heart rate ≥110/min | 20 |
| Systolic blood pressure <100 mmHg | 30 |
| Respiratory rate ≥30/min | 20 |
| Temperature <36° Celsius | 20 |
| Altered mental status | 60 |
| Arterial oxygen saturation <90 % | 20 |
| **sPESI** |  |
| Age >80 years | 1 |
| History of cancer | 1 |
| Chronic cardiopulmonary disease | 1 |
| Heart rate ≥110/min | 1 |
| Systolic blood pressure <100 mmHg | 1 |
| Arterial oxyhemoglobin saturation <90 % | 1 |
| **ICU-sPESI add-ons on sPESI** |  |
| **(I)** **I**ntubation status | 1 |
| **(C)** **C**onfusion/altered mental status | 1 |
| **(U)** **U**se of vasopressors/inotropes | 1 |
