# Supplementary material for: Predicting Hospital Survival in Patients Admitted to ICU with Pulmonary Embolism
Source: J Intensive Care Med. 2023 Nov 15;39(5):455–64. doi: 10.1177/08850666231212875 (PMC10935623; doi:10.1177/08850666231212875)
Supplement: sj-docx-4-jic-10.1177_08850666231212875 - Supplemental material for Predicting Hospital Survival in Patients Admitted to ICU with Pulmonary Embolism [file sj-docx-4-jic-10.1177_08850666231212875.docx]

**Supplementary Table 2.** Additional details of comorbidities prior to intensive care unit admission of patients with pulmonary embolism who required intensive care unit admission.

| **Comorbidities** | **All patients**  **N = 1,424** | **Survivors**  **N = 1,334** | **Non-survivors**  **N = 90** | **P**  **values** |
| --- | --- | --- | --- | --- |
| **COPD ^a^** |  |  |  | 0.007 |
| Mild | 55 (3.9%) | 50 (3.7%) | 5 (5.6%) |  |
| Moderate | 79 (5.5%) | 71 (5.3%) | 8 (8.9%) |  |
| Severe | 30 (2.1%) | 24 (1.8%) | 6 (6.7%) |  |
| **Cardiac dysrhythmias** |  |  |  | 0.374 |
| Atrial fibrillation | 112 (7.9%) | 102 (7.6%) | 10 (11.1%) |  |
| Other | 9 (0.6%) | 9 (0.7%) | 0 (0%) |  |
| **Cancer history** |  |  |  | <0.001 |
| Genitourinary | 57 (4.0%) | 52 (3.9%) | 5 (5.6%) |  |
| GI & Hepatobiliary | 50 (3.5%) | 44 (3.4%) | 6 (6.7%) |  |
| Respiratory | 37 (2.6%) | 28 (2.1%) | 9 (10.0%) |  |
| Breast | 37 (2.6%) | 34 (2.5%) | 3 (3.3%) |  |
| Other | 65 (4.6%) | 57 (4.3%) | 8 (8.9%) |  |
| **Diabetes mellitus** |  |  |  | 0.257 |
| No treatment | 44 (3.1%) | 38 (2.8%) | 6 (6.7%) |  |
| Antidiabetic medications only | 135 (9.5%) | 126 (9.4%) | 9 (10.0%) |  |
| Insulin dependent | 105 (7.4%) | 99 (7.4%) | 6 (6.7%) |  |
| **Renal failure** |  |  |  | 0.476 |
| Without dialysis | 12 (0.8%) | 11 (0.8%) | 1 (1.1%) |  |
| With dialysis | 13 (0.9%) | 12 (0.9%) | 1 (1.1%) |  |
| **Prior pulmonary embolism** |  |  |  | 0.731 |
| Single | 114 (8.0%) | 109 (8.2%) | 5 (5.6%) |  |
| Multiple | 8 (0.6%) | 8 (0.6%) | 0 (0%) |  |

Binary variables are count (percentage), continuous variables are median [IQR]. Individual features had no missing values.

^a^ Reported as “mild”, “moderate” or “severe” in the eICU database.

*Abbreviations*: COPD, chronic obstructive pulmonary disease; GI, gastrointestinal;
