# Supplementary material for: Predicting Hospital Survival in Patients Admitted to ICU with Pulmonary Embolism
Source: J Intensive Care Med. 2023 Nov 15;39(5):455–64. doi: 10.1177/08850666231212875 (PMC10935623; doi:10.1177/08850666231212875)
Supplement: sj-docx-5-jic-10.1177_08850666231212875 - Supplemental material for Predicting Hospital Survival in Patients Admitted to ICU with Pulmonary Embolism [file sj-docx-5-jic-10.1177_08850666231212875.docx]

**Supplementary Table 3.** Pulmonary Embolism Severity Index (PESI), Simplified Pulmonary Embolism Severity Index (sPESI) and ICU-modified Simplified Pulmonary Embolism Severity Index (ICU-sPESI) score components.

| **Score components** | **All patients**  **N = 1,424** | | **Survivors**  **N = 1,334** | | **Non-survivors**  **N = 90** | | **P values** | |  |
| --- | --- | --- | --- | --- | --- | --- | --- | --- | --- |
| **Demographics and comorbidities** | | |  | |  | |  | |  |
| Age, years ^a^ | 63.0 [51.0-75.0] | | 62.0 [51.0-74.0] | | 70.0 [59.2-80.8] | | < 0.001 | |  |
| Age > 80 years ^b^ | 207 (14.5%) | | 184 (13.8%) | | 23 (25.6%) | | 0.004 | |  |
| Sex (male) ^a^ | 710 (49.9%) | | 670 (50.2%) | | 40 (44.4%) | | 0.341 | |  |
| Chronic lung disease ^a^ | 254 (17.8%) | | 228 (17.1%) | | 26 (28.9%) | | 0.007 | |  |
| Heart failure ^a^ | 116 (8.1%) | | 108 (8.1%) | | 8 (8.9%) | | 0.947 | |  |
| Chronic CP disease ^b^ | 394 (27.7%) | | 360 (27.0%) | | 34 (37.8%) | | 0.036 | |  |
| History of cancer ^a,b^ | 246 (17.3%) | | 215 (16.1%) | | 31 (34.4%) | | < 0.001 | |  |
| **Vitals and treatments** | | | | |  | |  | |  |
| Heart rate ≥ 110 ^a,b^ | | 715 (50.2%) | | 647 (48.5%) | | 68 (75.6%) | | < 0.001 | |
| SBP < 100 mmHg ^a,b^ | | 794 (55.8%) | | 723 (54.2%) | | 71 (78.9%) | | < 0.001 | |
| Temperature < 36.0 °C ^a^ | | 130 (9.1%) | | 106 (7.9%) | | 24 (26.7%) | | < 0.001 | |
| Respiratory rate ≥ 30 ^a^ | | 743 (52.2%) | | 673 (50.4%) | | 70 (77.8%) | | < 0.001 | |
| SaO2 % <90 ^a,b^ | | 322 (22.6.1%) | | 270 (20.2%) | | 52 (57.8%) | | < 0.001 | |
| Altered mental status ^a,c^ | | 199 (14.0%) | | 156 (11.7%) | | 43 (47.8%) | | < 0.001 | |
| Intubated ^c^ | | 119 (8.4%) | | 79 (5.9%) | | 40 (44.4%) | | < 0.001 | |
| Vasopressors/inotropes ^c,d^ | | 79 (5.5%) | | 53 (4.0%) | | 26 (28.9%) | | < 0.001 | |

Data are count (percentage) or median [IQR].

Vital signs and treatments are obtained within the 24 hours of admission to ICU.

^a^ Included in the PESI.

^b^ Included in the sPESI.

^c^ Included in the *ICU-sPESI*.

^d^ Include vasoconstrictors and or inotropic infusions.

*Abbreviations*: SBP, systolic blood pressure; CP, cardiopulmonary; SaO2 %, percentage of oxyhemoglobin saturation; °C, degrees Celsius.
