# Supplementary material for: Predicting Hospital Survival in Patients Admitted to ICU with Pulmonary Embolism
Source: J Intensive Care Med. 2023 Nov 15;39(5):455–64. doi: 10.1177/08850666231212875 (PMC10935623; doi:10.1177/08850666231212875)
Supplement: sj-docx-6-jic-10.1177_08850666231212875 - Supplemental material for Predicting Hospital Survival in Patients Admitted to ICU with Pulmonary Embolism [file sj-docx-6-jic-10.1177_08850666231212875.docx]

**Supplementary Table 4.** Additional details of clinical variables obtained during the first 24 hours in the intensive care unit (details of the individual score components in the first 24 hours after admission are presented in Supplementary Table 3).

| **Clinical Variables** | **All patients**  **N = 1,424** | **Survivors**  **N = 1,334** | **Non-survivors**  **N = 90** | **P values** |
| --- | --- | --- | --- | --- |
| **Glasgow coma scale** (0-15) | 15.0 [15.0-15.0] | 15.0 [15.0-15.0] | 15.0 [8.0-15.0] | < 0.001 |
| Eye opening response | 4.0 [4.0-4.0] | 4.0 [4.0-4.0] | 4.0 [2.0-4.0] | < 0.001 |
| Motor response | 6.0 [6.0-6.0] | 6.0 [6.0-6.0] | 6.0 [5.0-6.0] | < 0.001 |
| Verbal response | 5.0 [5.0-5.0] | 5.0 [5.0-5.0] | 5.0 [1.0-5.0] | < 0.001 |
| **Vitals signs ^a^** |  |  |  |  |
| Respiratory rate, per min | 20.5 [18.0-23.5] | 20.5 [18.0-23.2] | 23.5 [19.7-27.5] | < 0.001 |
| SaO_2_, % | 96.5 [95.0-98.0] | 96.5 [95.0-98.0] | 97.0 [94.9-98.3] | 0.691 |
| Heart rate, beats per min | 88.0 [76.5-98.0] | 87.0 [76.0-97.2] | 97.6 [85.8-107.9] | < 0.001 |
| Systolic BP, mmHg | 120.0 [108.5-131.0] | 120.0 [109.5-131.5] | 109.6 [97.2-121.9] | < 0.001 |
| Mean BP, mmHg | 84.0 [75.5-92.0] | 84.0 [76.0-92.0] | 73.8 [66.6-85.0] | < 0.001 |
| Diastolic BP, mmHg | 69.5 [62.0-77.5] | 70.0 [62.4-78.0] | 60.0 [55.1-69.4] | < 0.001 |
| Temperature, °C | 36.8 [36.6-37.0] | 36.8 [36.6-37.0] | 36.6 [36.3-36.9] | < 0.001 |

Binary variables and categories are count (percentage), continuous variables are median [IQR]. Vital signs had <1.5% missing values except for the respiratory rate with 7.4% and temperature with 3.0% missing values. All other features had no missing values.

^a^ Median values

*Abbreviations*: SaO2 %, percentage of oxyhemoglobin saturation; BP, blood pressure; °C, degrees Celsius.
