# Supplementary material for: Predicting Hospital Survival in Patients Admitted to ICU with Pulmonary Embolism
Source: J Intensive Care Med. 2023 Nov 15;39(5):455–64. doi: 10.1177/08850666231212875 (PMC10935623; doi:10.1177/08850666231212875)
Supplement: sj-docx-7-jic-10.1177_08850666231212875 - Supplemental material for Predicting Hospital Survival in Patients Admitted to ICU with Pulmonary Embolism [file sj-docx-7-jic-10.1177_08850666231212875.docx]

**Supplementary Table 5.** Sensitivity analyses of the area under the receiver operating characteristic (AUROC) at different time intervals following admission to the intensive care unit with pulmonary embolism.

| **Time-interval from admission**  (hours) | **AUROC** | | | **P-values** | | |
| --- | --- | --- | --- | --- | --- | --- |
|  | **PESI** | **sPESI** | **ICU-sPESI** | PESI vs sPESI | sPESI vs ICU-sPESI | PESI vs. ICU-sPESI |
| **2** | 0.819 | 0.746 | 0.803 | **0.013** | **0.005** | 0.522 |
| **3** | 0.832 | 0.761 | 0.808 | **0.012** | **0.013** | 0.321 |
| **6** | 0.842 | 0.753 | 0.816 | **0.002** | **0.002** | 0.255 |
| **12** | 0.841 | 0.746 | 0.816 | **0.001** | **<0.001** | 0.290 |
| **24** | 0.848 | 0.777 | 0.847 | **0.010** | **<0.001** | 0.945 |

All variables (both the vitals and the 3 “ICU”-variables of the ICU-sPESI) are adjusted according to the timeframe following admission to the intensive care unit. Bold text highlights significant comparisons.
